# Effectiveness and Recommendations for the Use of Dental Masks in the Prevention of COVID-19: A Literature Review

**DOI:** 10.1017/dmp.2020.255

**Published:** 2020-07-17

**Authors:** Josabet Johana Arellano-Cotrina, Nicole Marengo-Coronel, Katherine Joselyn Atoche-Socola, Claudio Peña-Soto, Luis Ernesto Arriola-Guillén

**Affiliations:** School of Dentistry, Universidad Científica del Sur, Lima, Peru; Division of Oral Rehabilitation, School of Dentistry, Universidad Científica del Sur, Lima, Peru; Division of Periodontology, School of Dentistry, Universidad Científica del Sur, Lima, Peru; Division of Orthodontics, School of Dentistry, Universidad Científica del Sur, Lima, Peru

**Keywords:** COVID-19, dentistry, face masks, FFP2, N95, respirator

## Abstract

The purpose of this investigation was to identify, synthesize, and compare all the current information on the efficacy of dental masks, emphasizing their use, types, and filters to prevent the spread and infection of COVID-19 and other infectious diseases. A bibliographic search of the main scientific databases was carried out using the words “masks, COVID-19, and dentistry.” Articles without language restriction up to May 31, 2020, were obtained. The types of masks, their half-life, method to use, sterilization, and proposed alternatives for dental masks were analyzed. Most of the articles refer to the use of N95 or FFP2 respirators presented as a strategy to extend the life of the masks and limited reuse. Regarding sterilization, most of the articles presented studies using ultraviolet germicidal irradiation as the sterilization method. Regarding respirator mask half-life, we recommend prolonged use, combined with a disposable surgical mask over the respirator mask. Finally, the use of N95 or FFP2 respirators are recommended as part of personal protective equipment for dental use.

As health professionals, dentists are in the front line and have a high risk of contracting infectious diseases, which can be transmitted by direct or indirect contact through instruments or body fluids, such as blood and saliva.^1^ Therefore, dentists must comply with biosafety standards to protect both themselves and their patients.^2^ Safety in dental care should be similar for all patients and not only for those with infectious diseases. Operators and patients may be potential asymptomatic carriers of different microorganisms, causing cross-infections that can affect anyone in dental care and that can be transmitted to the family environment, increasing the risk of contagion.^3^

On December 31, 2019, the authorities of Wuhan, China, notified the World Health Organization (WHO) of the presence of an outbreak of viral pneumonia of unknown origin, mainly among vendors or operators of the Marine Huanan food market.^4-6^ Within a few days the disease was called coronavirus disease 2019 (COVID-19) caused by the severe acute respiratory syndrome coronavirus 2 (SARS-CoV-2 virus, which is transmitted mainly by close contact with secretions or excretions (droplets) of infected patients in the absence of the necessary protective barriers.^7^ Thus, COVID-19 spread rapidly to different parts of the world, and on March 11, 2020, COVID-19 was categorized as a global pandemic by the WHO,^5,8^ the main symptoms being high fever (83-98%), dry cough (76-82%), and difficulty in breathing (17-29%).^4,9^

To ensure good clinical practice, health professionals must comply with a set of rules and behaviors to reduce the risk of contracting infections through the use of protective barriers in each procedure, consisting in the use of scrubs, gowns, hats, disposable gloves, protective glasses, and masks.^10,11^ According to the manufacturers, surgical masks must achieve efficient filtration, resistance to fluids, pressure differential, and flammability. These masks must create a hermetic seal against the skin, preventing the passage of particles, such as aerosols or splashes that may contain bacteria or viruses. Mask quality certification relies on 2 types of tests to assess filtration efficiency, including quantitative and qualitative tests, such as the particulate filtration efficiency (PFE) test and the bacterial filtration efficiency (BFE) test.^11,12^ Obtention of this certification has a direct impact on the biosafety of professionals and patients. However, effective prevention of infectious diseases depends on the type of mask used.

Fear of the spread of serious respiratory diseases persists, as in the case of the recent coronavirus pandemic and is largely due to the current lack of effective antiviral drugs and vaccines.^13,14^ The WHO and the United States Centers for Disease Control and Prevention (CDC) recommend a series of fundamental preventive measures, such as protective equipment for health personnel during the care of patients with suspected or confirmed COVID-19, one of the most effective being masks. However, the WHO suggests their use only in the presence of symptoms, and the CDC indicates that the use of N95 respirators is exclusively for health personnel and not for the general public.^15^ These masks play an important role in the control of the spread of aerosols in the case of coughing, talking, or sneezing.^16,17^ Therefore, the purpose of this research was to identify and synthesize all the current information comparing the efficacy of dental masks, to increase our knowledge about the correct use of different types of masks and filters to prevent the spread and contagion of the COVID-19 virus and other infectious diseases.

## METHODS

A bibliographic search was carried out in the main databases of the international scientific literature on health sciences (MEDLINE) by means of PubMed, EBSCO, SCOPUS, SCIELO, and the Latin American and Caribbean Literature in Health Sciences (LILACS), using the following keywords: masks, COVID-19, and dentistry. Articles without language restriction up to May 31, 2020, were obtained. Experimental studies, literature review articles, and systematic reviews were used and opinion articles, letters to the editor, and editorials were excluded (Table 1).

**TABLE 1:** Search Strategies in the Main Databases

Database	Keywords
PubMed	((((((dentistry[MeSH Terms]) OR (dentistry)) AND (covid 19)) AND (mask)(((dentistry[MeSH Terms]) OR (dentistry)) AND (coronavirus)) AND (N95)) (((dentistry[MeSH Terms]) OR (dentistry)) AND (covid 19)) AND (respirator)) ((covid 19[MeSH Terms]) OR (covid 19)) AND (facemask)) (((covid 19[MeSH Terms]) OR (covid 19)) AND (FFP2)) OR (N95)
SCOPUS	(TITLE-ABS-KEY (COVID-19) OR TITLE-ABS-KEY (dentistry))
EBSCO	(coronavirus or COVID19) AND (dentistry or dental) AND (mask or N95 or FFP2)
SCIELO	(covid 19) AND (dentistry) OR (((covid 19) AND (dentistry)) AND (mask)) OR (n95)
LILACS	(tw:(COVID-19)) OR (tw:(coronavirus 2019)) OR (tw:(mask)) OR (tw:(N95)) OR (tw:(FFP2))

### Types of Masks

When a pandemic begins difficulties arise due to the lack of vaccines and ideal treatments for the disease, and, therefore, protection barriers play an important role in controlling the spread and prevention of the disease.^18^ Taking this into account, 2 types of masks have been described: surgical and conventional (or respiratory). The efficacy of these masks depends on their structure and filtering capacity. In this sense, respiratory masks guarantee better protection compared with surgical masks, both of which are disposable.^19^

Under the European standard, surgical masks are considered a medical device with an official nomenclature of the EN 14683 standard that classifies these masks as Type I, Type II, and IIR. The latter is classified as the most effective for presenting a microbial barrier and resistance to splashes, offering a filtration rate of around 80%.^18,20^ They are designed for protection in only 1 direction to avoid the transmission of infectious agents carried by the user. They prevent the passage of microorganisms present from the inside out; therefore, the use of these masks is recommended for COVID-19 patients.^19,21,22^ However, these surgical masks do not ensure a good hermetic seal, and thereby allow particles to enter around the edges.

The classification of respiratory masks is independently certified by 2 major entities: the European Committee for Standardization (EN) and the National Institute for Occupational Safety and Health (NIOSH). Both entities guarantee a percentage of filtering capacity of particles that measure 0.3 microns in diameter. Respiratory masks must have multiple layers of polypropylene and electrostatic charge, providing adequate protection in 2 directions; that is, they are able to filter both incoming and outgoing air and must be resistant to liquid spray, blood splatter, and of other bodily fluids. Likewise, masks are considered to be effectively adjusted when a hermetic seal is achieved on contact with the skin.^17,19,21,23,24^

The European standard EN 149: 2001 establishes 3 categories or levels of protection for respiratory masks according to their filtering facepiece (FFP) parts, and these are divided into FFP1, FFP2, and FFP3, with a particle filtration capacity of 0.3 microns of 80%, 95%, and 99%, respectively.^19,20^ On the other hand, the NIOSH establishes 9 respirator classifications, all with a particulate filtering capacity combining the respirator series (N, R, or P) and the level of efficacy (95%, 99%, 100%). The first part of the respirator classification indicates the resistance of the filter to degradation when exposed to oil-based aerosols, whereas the N series is for use in particulate environments and oil-free aerosols, and the R and P series are for use in particulate environments with and without oil. The number determines the filtering capacity of 0.3-micron particles, being measured in percentages of 95%, 99%, and 100%.^17,23,25^

The use of N95 masks has been considered a US standard administered by the NIOSH. These masks are designed to protect users from air particles, including aerosols,^26^ with a particle filtration capacity of 0.3 microns of 95% and have less leakage in the face seal due to the tight fit to the user’s face^19,22,24,27,28^ N99 masks have a 0.3-micron particle filtration capacity of 99%, while N100 masks provide 99.7% filtration protection.^23^

On the other hand, the use of KN95 respirators of Chinese origin are available in the dental market and comply with GB 2626-2006 regulations. These masks have a filtration capacity of 94-95% of particles with 4 overlapping layers, which are fused together to avoid the exit of particles from the carrier and the aspiration of aerosols or drops that may contain the virus. These respirators are considered to be functionally similar to the NIOSH-certified N series.^12,23^

### Mean Life of the Masks

An important approach to moderating mask wear and avoiding scarcity is to adopt strategies for mask reuse.^17,18^ The CDC recommends 2 conservation strategies for respirators: extended use and limited reuse.^29,30^

Surgical and respiratory masks are for single use per patient. However, conservation of these resources is imperative in this crisis. Thus, an alternative is use with multiple patients,^12,20^ but with strict biosecurity conditions, involving safeguards, evaluation of sealing, and mask integrity during use. A second surgical mask can be used on the respiratory mask to serve as direct protection against patient fluids, being discarded after use.

In the case of using only 1 respiratory mask while under great exposure to infectious droplets due to the aerosols, it is not recommended to reuse the same mask between patients because of a higher risk of contamination. However, this condition can be ameliorated with the use of a face protection mask or the use of 2 masks, which will allow the second protective mask to be discarded and the facial mask disinfected, thereby preserving the respiratory mask.^12,20^

The second form of conservation of respiratory masks is limited reuse, which is the removal of the mask after each patient with restrictions limiting the number of uses. However, this requires strict validation regarding cleaning, sterilization, and functional performance.^29^ It is important to consider that the duration of use for surgical masks should not exceed 4 h and 8 h for FFP masks.^19,20,31^

In the literature, few studies have evaluated the reuse of masks. One study evaluated health policies of 27 countries (in Europe and the Americas), finding that widespread use and limited reuse of masks is allowed in 10 countries. On the other hand, more than 60% of the countries do not recommend 1 of these 2 strategies.^30^

### How to Use

In 1 of its publications, the CDC recommends how to use and dispose of respiratory masks.^32^

#### Placement

Secure the ties or elastic bands to the middle of the back of the head and neck, and then adjust the band to the bridge of the nose, around the face and chin. Finally, check the fit of the mask. After placement, hands must be disinfected with alcohol and washed with soap for at least 20 s.

#### Removal

The front of the mask is contaminated and should not be touched. Hold the ties or elastic bands of the mask and remove them upward without touching the front. Discard in the indicated garbage container and wash your hands with soap for at least 20 s.

### Sterilization

Given the shortage of personal protective equipment (PPE), sterilization and disinfection methods have been determined to prolong the effectiveness of the masks for the prevention of virus transmission. It is important that the sterilization treatment does not deteriorate the material of the respiratory mask, which would decrease the filtering power against infectious pathogens. The CDC has recommended different chemical, radioactive, and physical sterilization methods.^24^

Different decontamination strategies, such as sterilization by exposure to ultraviolet germicidal irradiation (UVGI), ethylene oxide, or vaporized hydrogen peroxide, have been shown to be effective in maintaining adequate protective function.^30,33-36^

In 1 study, different common sterilization methods for masks were performed including heat treatment, cabinet UVGI sterilizer, steam, alcohol solutions, and chlorine-based solutions. The last 2 methods caused degradation of the static charge required by the FFR. On the other hand, dry heat in the presence of humidity at 100°C preserved the characteristics of the N95 respirator, limiting temperature and humidity for 30 min. This study determined that the best sterilization method was UVGI (254 nm, 8W) with 3.6 J/cm^2^.^17^

UVGI uses UV-C radiation to inactivate microorganisms causing damage to deoxyribonucleic acid (DNA), preventing replication. Several studies have demonstrated the efficacy of UVGI in reducing contamination by drops applied to the N95 respirator, with effective destruction of SARS-CoV and Middle East respiratory syndrome coronavirus (MERS-CoV)^24,37,38^ However, the use of UVGI for the destruction of this new virus requires further studies to establish the exact exposure or light intensity required, as 3M reports have shown that UVGI treatments damage the mask, while other studies show that this technique had no impact on the masks.^17^

Combination strategies have been studied, including the use a solution of sodium hypochlorite with microwave-generated steam UVGI, demonstrating effective sterilization with a reduction of multiple registrations of MS2 bacteriophage virus from masks. This virus is 7-10 times more resistant to aerosolization and ultraviolet light than the coronavirus.^39^

The susceptibility of the virus to gamma irradiation has shown good disinfecting ability by penetration of all the layers of the respirators. However, the use of ionizing radiation is limited because gamma radiation cannot be performed in a health-care center, requiring the need for transportation to another location entailing a risk for the personnel transporting the masks.^29^

On the other hand, the CDC reports that some autoclave methods at 160°C in dry heat, 70-75% isopropyl alcohol and soapy water can deteriorate the filter of the respirators, and consequently allow the access of particles through the mask.^24^

For mask sterilization, certain requirements must be taken into account, such as the efficacy against the SARS-CoV2 organism, avoiding damage to the respirator filtration changes in the physical characteristics of the respirators, and ensuring biosafety to the persons who must wear the respirator mask.^29^

### Proposed Alternatives of Masks for Dentists

According to the information obtained from COVID-19, the transmission routes are direct or indirect contact with contaminated patients, saliva drops,^12^ and large aerosol particles suspended in the air (up to 1 m away), which remain present for a short period of time.^17,21,40^ In dentistry, there is direct contact with the patient through fluids, such as blood or saliva, and many dental treatments generate aerosols. It should also be taken into account that the SARS-CoV2 virus has affinity for the angiotensin 2 converting enzyme receptor (ACE-2), which is found in the respiratory tract and the salivary gland ducts, producing a high viral load in saliva.^12,17^

Different models of masks are available, making it difficult to choose the most suitable type of respirator for dental care. Therefore, according to past recommendations, it is recommended to perform fit tests of masks for health personnel to determine the best fit and hermetic seal according to facial dimensions, ethnic origin, and appearance of the fit.

The literature recommends that surgical masks should not be used as a substitute for respiratory masks.^19,27,41^ The use of an N95, FFP2, and FFP3 mask is recommended for personnel working with aerosols. Filtration is achieved by combining a polypropylene network and electrostatic charge,^21^ thereby explaining their good protective effect against aerosols, and the reason why respirators should not be used by the general public.^22,31,42^

On analyzing the filtering capacity according to the mask classification, the N95 has shown a similar filtration efficacy to that of FFP2 or KN95.^24^ Regarding mask sterilization, although it is necessary to determine the exact UVGI doses for mask sterilization against the SARS-CoV-2 virus, this strategy provides a possibility to extend the use of the limited supply of respirator masks against COVID-19, being both profitable and accessible.^33^

A major problem with respirator masks is discomfort and the generation of humidity inside, decreasing air permeability. Therefore, a super absorbent polymer (SAP) has been designed for use in respirator masks. This harmless material absorbs large amounts of liquid is used in baby diapers, sanitary napkins, and incontinence pads. An absorbent layer or SAP pad is cut according to the shape and size of the respirator and finally placed inside the respirator. The SAP pad helps to quickly absorb exhaled moisture, leading to a longer mask life cycle and providing greater comfort to the professional.^43^

## DISCUSSION

In these times of international health alert, dentists have a high risk of contracting COVID-19 due to direct contact with body fluids during patient care. Therefore, the use of PPE is extremely important for the protection of dentists, with masks being part of the clothing for daily use to avoid aspiration of virus particles.^44^ The aim of this literature review was to identify and synthesize all the current information available on the efficacy of the masks in dentistry to prevent the spread of the new COVID-19.

Currently the different types of masks in the market cause confusion at the time to choosing the most adequate equipment. There are 2 types of masks: surgical masks and respiratory masks. Surgical masks have a filtering capacity of 80% of particles compared with N95 and FFP2 respirators, which have a 95% filtering capacity of particles measuring 0.3 microns in diameter due to their multiple layers of polypropylene in combination with an electrostatic charge. This good filtering capacity has led to recommending their use a global standardization.^12,17,19,24,27^

Ideally, 1 mask is used per patient. However, regulatory authorities are trying to take action involving the widespread use and limited reuse of masks due to the global shortage of PPE. One of the most recommended strategies is the prolonged use of the respirator for different patients, using a second surgical mask over the respirator to protect it from fluids to preserve its integrity. However, it is highlighted that this strategy is only a resource for the protection of health personnel during the COVID-19 pandemic, due to the shortage of this equipment.^20,30^

Regarding the mask sterilization method, many studies report that COVID-19 is susceptible to sterilization by UVGI, which inactivates these microorganisms by damaging their DNA.^17,29^ Likewise, this method has shown great efficacy, maintaining the protective function of the mask and filtering power against pathogens. In the same way, hydrogen peroxide vapor sterilization is an effective method to prolong mask efficacy. However, these methods still require further study to ensure preservation of the physical characteristics of the respirators and adequate biosecurity for dentists.^30,33-39^

There are different studies of the efficacy and recommendations for the use of masks to prevent the spread of COVID-19.^19,21,30^ The information described in this study highlights the importance of the resistance of the masks to droplets and aerosols for the prevention of inhalation of contaminating particles, guaranteeing biosecurity to dentists. Recommendations are based on the largest evidence available. On the other hand, there are several limitations regarding sterilization for reuse of respirators that should be recognized. Further studies are required to standardize adequate and effective exposure to prolong the efficacy of respirator masks. Likewise, it is recommended that dental centers adhere to the correct use of respirators, otherwise, they risk endangering their health and the transmission of COVID-19 to those in their work environment.

## CONCLUSIONS

The use of N95 or FFP2 respirators is recommended as part of PPE for dental use during patient care. For a longer useful life of respiratory masks, it is recommended to add a surgical mask together with the use of a face mask. Likewise, at the time of placing the mask, it is important to ensure a correct fit and hermetic seal against the skin. Likewise, at the time of removal, avoid direct contact with the external part of the mask. Finally, as a method of mask sterilization, up to now the use of UVGI, hydrogen peroxide steam, and heat guarantee the preservation of the filtering and structural capacity of masks, providing dentists with adequate protection. Nonetheless, more studies are needed for more information on the exact doses of UVGI to implement.
